# The Emerging Role of “Failure to Rescue” as the Primary Quality Metric for Cardiovascular Surgery and Critical Care

**DOI:** 10.3390/jcm12144876

**Published:** 2023-07-24

**Authors:** Dimitrios E. Magouliotis, Andrew Xanthopoulos, Prokopis-Andreas Zotos, Arian Arjomandi Rad, Evangelos Tatsios, Metaxia Bareka, Alexandros Briasoulis, Filippos Triposkiadis, John Skoularigis, Thanos Athanasiou

**Affiliations:** 1 Unit of Quality Improvement, Department of Cardiothoracic Surgery, University of Thessaly, Biopolis, 41110 Larissa, Greece; 2 Department of Cardiology, University of Thessaly, Biopolis, 41110 Larissa, Greece; andrewvxanth@gmail.com (A.X.); ftriposkiadis@gmail.com (F.T.); iskoular@gmail.com (J.S.); 3 Department of Cardiothoracic Surgery, University of Thessaly, Biopolis, 41110 Larissa, Greece; zotospro@hotmail.com (P.-A.Z.); evangelos.tats@gmail.com (E.T.); 4 Department of Surgery and Cancer, Imperial College London, St Mary’s Hospital, London W2 1NY, UK; arian.arjomandi-rad16@imperial.ac.uk (A.A.R.); t.athanasiou@imperial.ac.uk (T.A.); 5 Department of Anesthesiology, University of Thessaly, Biopolis, 41110 Larissa, Greece; barekametaxia@hotmail.com; 6 Department of Therapeutics, Faculty of Medicine, National and Kapodistrian University of Athens, 10679 Athens, Greece; alexbriasoulis@gmail.com

**Keywords:** failure to rescue, FTR, cardiac surgery, cardiology, cardiovascular, mortality, quality

## Abstract

We conducted a thorough literature review on the emerging role of failure to rescue (FTR) as a quality metric for cardiovascular surgery and critical care. For this purpose, we identified all original research studies assessing the implementation of FTR in cardiovascular surgery and critical care from 1992 to 2023. All included studies were evaluated for their quality. Although all studies defined FTR as mortality after a surgical complication, a high heterogeneity has been reported among studies regarding the included complications. There are certain factors that affect the FTR, divided into hospital- and patient-related factors. The identification of these factors allowed us to build a stepwise roadmap to reduce the FTR rate. Recently, FTR has further evolved as a metric to assess morbidity instead of mortality, while being also evaluated in the context of interventional cardiology. All these advances are further discussed in the current review, thus providing all the necessary information to surgeons, anesthesiologists, and physicians willing to implement FTR as a metric of quality in their establishment.

## 1. Introduction

Cardiac surgery as a discipline, along with its surgical community, has always been at the forefront of measuring, assessing, and improving the quality of provided healthcare services. At the core of these efforts has always been the creation, validation, and adoption of novel quality metrics to fulfill all the previously mentioned purposes. Based on the generally excellent outcomes of cardiac surgical operations, the use of mortality as a metric of quality was rapidly considered inadequate for providing accurate insight into clinical outcomes, given the low mortality rate and relative safety of these procedures [1]. In fact, the focus of research gradually diverged towards enhancing our understanding of surgical mortality, considered as a direct consequence of severe complications related, to a certain extent, to hospital rather than patient factors [2]. In this context, Silber introduced, in 1992, the concept of failure to rescue (FTR), defined as death during an in-hospital stay among patients who presented a postoperative complication [2]. Despite the slow adoption of FTR, the concept has gained increasing attention in all fields of surgery. Following its adoption in clinical practice, a number of studies were conducted to assess its value and demonstrated that departments with the lowest perioperative mortality did not necessarily have the lowest incidence of complications [2,3]. Instead, differences in FTR rates demonstrated that these successful departments were more ready to efficiently manage such complications [2,3,4]. Consequently, it is no wonder that FTR was adopted early as a patient-safety and quality metric by several bodies and agencies, such as the Agency for Healthcare Quality and Research (AHQR) [5], the National Quality Forum [6], the Centers for Medicare and Medicaid Services [7], and the Society of Thoracic Surgeons (STS) [8].

From its first adoption in cardiac surgery by Pasquali and colleagues [9] to its current state, there has been an increasing amount of evidence highlighting its value as an important quality metric [10]. Therefore, the purpose of the present study is to review the existing data in the literature regarding the role of FTR as a quality metric in cardiac surgery and cardiovascular critical care, thus providing the best, most up-to-date level of evidence currently available on the topic.

## 2. Materials and Methods

### 2.1. Search and Articles Selection Strategy

The current review was designed in accordance with the protocol agreed upon by all authors and the Preferred Reporting Items for Systematic Reviews and Meta-Analyses [11]. A systematic literature search was performed in two databases: (1) Scopus (ELSEVIER) and (2) Pubmed (Medline) (last date of literature search: 30 March 2023). The following terms were used in all possible combinations: “cardiac surgery”, “heart surgery”, “cardiovascular”, “cabg”, “coronary artery bypass grafting”, “aortic valve replacement”, “avr”, “failure to rescue”, “failure-to-rescue”, “ftr”, and “quality metric”. Inclusion criteria were (1) original reports with >10 patients, (2) published from 1992 to 2023, (3) written in English, (4) conducted on human subjects, and (5) reporting outcomes of patients undergoing cardiac surgery or being monitored and treated following a cardiac surgical operation. We excluded all duplicate articles and hand-searched the reference lists of all articles that were included for additional studies. Two independent reviewers (DEM, AΧ) extracted data from the included studies. Any potential discrepancies between the two investigators regarding the inclusion/exclusion of the selected studies were discussed with a senior author (TA) to incorporate only the articles that best matched the criteria until a consensus was reached. Regarding the FTR term, this was defined as any death during the postoperative in-hospital stay period among patients who underwent cardiac surgery and presented a postoperative complication.

### 2.2. Data Extraction and Quality Assessment

For every included study, we extracted data relative to the number, genders, and ages of the patients and the type of procedure, along with complication, mortality, and FTR rates. To evaluate the appropriateness of the included non-RCTs, we employed the Newcastle–Ottawa Scale (NOS) [12]. The scale uses a range varying from 0 to 9 stars, and studies with a score equal to or higher than five stars are considered to have an adequate methodological quality. No RCTs were identified/included regarding this topic. Two reviewers (DEM, AX) rated the studies independently and discrepancies were discussed until a consensus was reached.

## 3. Results

### 3.1. Search Strategy and Patient Demographics

**Figure 1:**
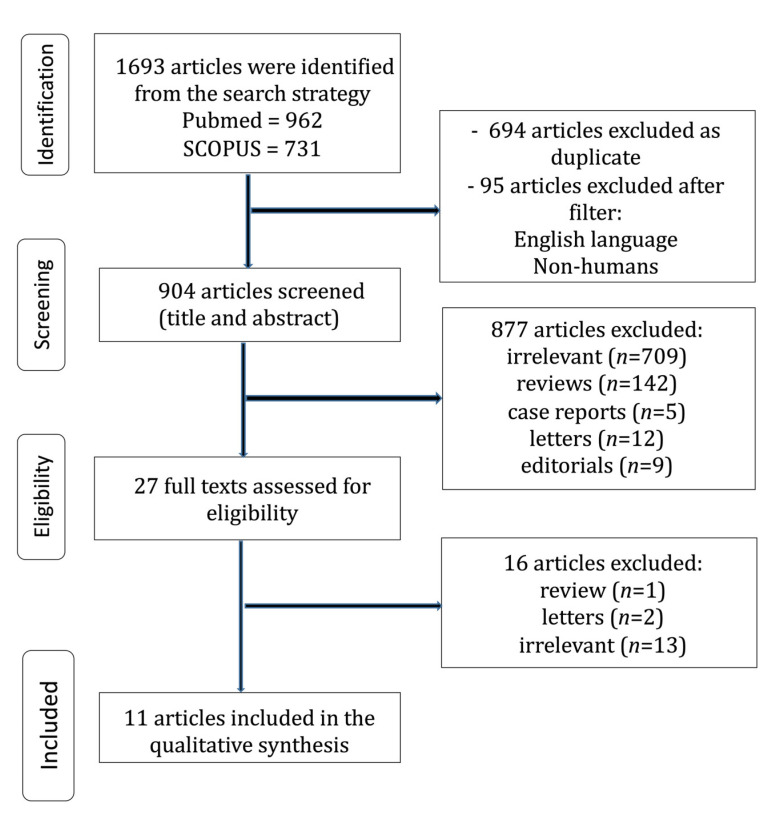
Trial flow of the current review.

**Figure 2:**
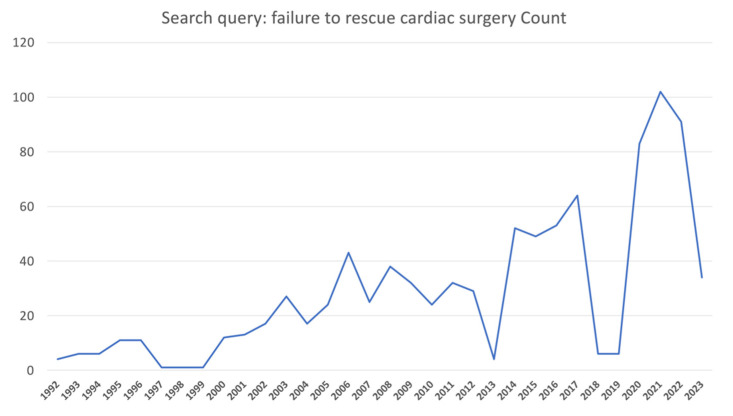
Article count per year in Pubmed/Medline for search topic: Failure to rescue in cardiac surgery. Only the first quarter of 2023 is included.

A flow diagram regarding the search strategy is provided in Figure 1, and the Prisma Checklist is demonstrated in Table S1. Characteristics of the incorporated studies are demonstrated in Table 1. Over the last three decades, there has been a great increase in published articles on the topic of FTR, as demonstrated in Figure 2. From the 1693 articles that were originally retrieved, eleven studies [1,13,14,15,16,17,18,19,20,21,22] were finally included in the present review. The level of agreement between the reviewers was “almost perfect” (kappa = 0.904; 95% CI: 0.782, 1.000). All studies were retrospective and four studies [1,16,17,20] implemented data from the STS database. The included studies were published between 2014 and 2022. The FTR rate ranged between 5.4% to 19.8% among the studies.

### 3.2. Evolution of the Definition of FTR 

**Figure 3:**
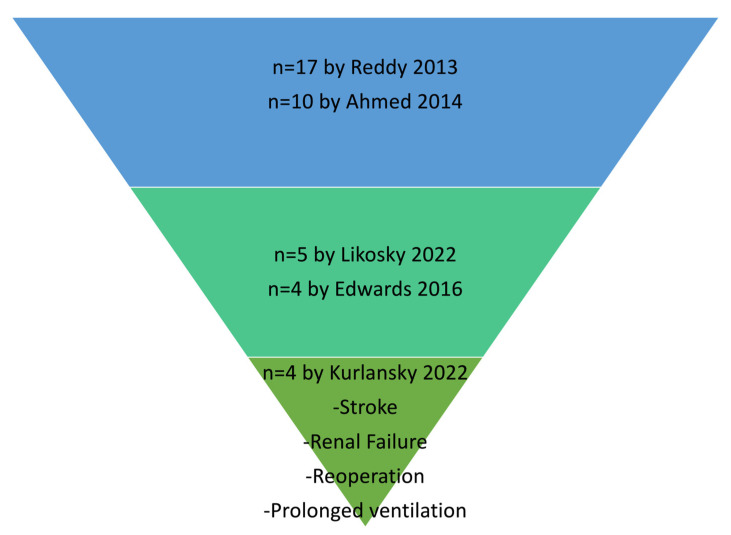
The evolution of the failure to rescue (FTR) metric to, finally, include four major complications. *n* = number. References: 1. Reddy 2013 [20]; 2. Ahmed 2014 [13]; 3. Likosky 2022 [18]; 4. Edwards 2016 [16]; Kurlansky 2022 [17].

The concept behind the definition of FTR in cardiac surgery has been greatly affected by the range of complications that have been evaluated and finally included in the definition of FTR. Based on the wide or narrow spectrum of assessed complications, the FTR rate has historically presented an important variation, as demonstrated in Figure 3. In this context, Ahmed et al. [13] defined FTR using the following ten complications: reoperation for bleeding or bleeding death, mediastinitis, respiratory failure, renal failure, septicemia, neurologic complications, cardiorespiratory arrest or arrhythmia, perioperative myocardial infarction, placement of a postoperative intra-aortic balloon pump, or sternal wound dehiscence. Following this definition, they reported an FTR rate of 19.8% [13]. In another study, Reddy and colleagues [20] also included a broad range of 17 complications. According to their outcomes [20] FTR rates ranged between 6.6% and 13.5% for low- and high-mortality centers. Nonetheless, more recent studies tend to assess FTR based solely on major complications. In their studies, Edwards et al. [16] and Likosky et al. [18] implemented just four and five major complications (stroke, surgical re-exploration, deep sternal wound infection, renal failure, prolonged intubation), respectively. Nonetheless, the heterogeneity regarding the definition of FTR and the selection of the complications of interest contributes to an important bias regarding the FTR metrics of different institutions.

In a recent report [17], Kurlansky et al. validated a new FTR quality metric focused on the institutional effectiveness of postoperative care. FTR was defined as death after one of four complications:

1. Stroke;
2. Renal failure (increase in serum creatinine levels over 2.0, or two times the preoperative creatinine level, or new dialysis);
3. Reoperation for any cause;
4. Prolonged ventilation (>24 h postoperatively).

The main advantage of this FTR definition is that incorporates only four variables, thus being relatively easy for achieving universal adoption and homogeneity among different institutions. Nonetheless, ideally, the FTR metric should focus on specific new complications that can cause mortality through an etiologic relation but also have the potential for successful management and patient rescue. For instance, these complications include cardiac arrhythmias, thrombosis (coronary, pulmonary, or vascular), cardiac/respiratory arrest bleeding or tamponade, and gastrointestinal ischemia or perforation. In this context, Strobel et al. recently proposed that cardiac arrest should be included in the FTR definition [23]. In addition, the inclusion of reoperation as part of the FTR metric is highly debatable. In fact, reoperation commonly represents a part of the rescue strategy for managing a complication and failure to reoperate would lead to mortality. Consequently, it is questionable whether it should be used as part of the FTR metric. Given the importance of the dilemmas that are posed, it is crucial to standardize the definition of FTR in cardiac surgery and cardiovascular intensive care, potentially through a Delfi approach consensus, to avoid comparing “apples with oranges” when evaluating quality outcomes.

### 3.3. Factors Affecting FTR

An in-depth understanding of the different factors that affect and contribute to success or failure in rescuing a patient who presents a complication following a cardiac surgical operation represents, perhaps, the first and most crucial step in implementing the FTR in clinical practice. In the following paragraphs, we try to outline the most important aspects in a stepwise manner, starting from the individual patient and reaching institution-level parameters.

### 3.4. Independent Patient Level

Clinical status and frailty level contribute to higher postoperative morbidity, mortality, and FTR rates. According to a study [14] that incorporated data from over a million cardiac surgery patients, frailty contributed to a higher FTR rate compared to that of non-frail patients. In fact, the group of frail patients were associated with an increased age and a higher incidence of heart failure and chronic lung, liver, and renal diseases [14]. Although its purpose is not to assess frailty status, perhaps the STS risk stratification tool [1] can highlight high-risk patient groups, thus providing an opportunity for increased awareness of their postoperative care needs.

### 3.5. Hospital Level

The first important aspect that affects FTR is the role of the institution and the department, as in whether it has an academic role or not. In an academic or teaching context, the most important characteristic is the presence of trainees. Nonetheless, there is contradictory evidence on their impact on clinical outcomes and FTR rates. In fact, there are certain studies that have demonstrated the beneficial role of trainees’ presence on a patient’s perioperative pathway [24,25]. Nonetheless, trainees must be adequately trained in order to be able to assess and identify a complication at an early stage and then escalate the treatment adequately by informing their supervisors. In cases where the level of the trainees’ education and experience is not adequate, a higher FTR rate is a potential outcome [2]. Such observations have led many institutions to adopt an intensivist consultation for the management of complex cases [25]. In fact, there is evidence demonstrating the superiority of intensivists over trainees in the management of cardiovascular patients at the ICU in terms of major postoperative complications [26].

Furthermore, the institutional staffing of physicians from allied disciplines and experienced nurses, along with allied support staff, plays a crucial role in the patient rescue plan. There are two main aspects that characterize the quality of hospital staffing. The first is the absolute physician-to-patient and nurse-to-patient ratios. The second is the expertise level of the staff. Both parameters highly contribute to the reduction of the FTR rate [2]. In this context, there is mounting evidence demonstrating a negative correlation between the nurse–patient ratio and FTR rate [2,25,26]. In other words, as the nurse-to-patient ratio increases, the FTR rate lowers. A possible explanation for this is that, in institutions with low physician- or nurse-to-patient ratios, the staff has the duty of caring for more patients, thus reducing their time dedicated to each patient and leading to an inability to detect patient deterioration early and escalate their level of care. In addition, a high patient burden contributes to poor communication among staff and job dissatisfaction, along with body and emotional exhaustion [26]. In addition to all the previously reported factors, the design and implementation of adequate nighttime/weekend physician/nurse shifts are critical in reducing FTR [26].

A final important aspect that affects FTR at the hospital level is the volume of cases that the center treats. There is growing evidence highlighting an association between high volumes of cases and lower FTR rates [27]. The primary argument in favor of high-volume centers is that they potentially have more standardized treatment pathways for perioperative care [27]. In this context, a study that incorporated data from 119,434 Medicare patients who underwent major cardiac surgery operations demonstrated similar complication rates between low- and high-volume centers, but a significantly higher FTR rate in low-volume centers [28].

### 3.6. Building a Stepwise Roadmap to Successful Rescue

**Figure 4:**
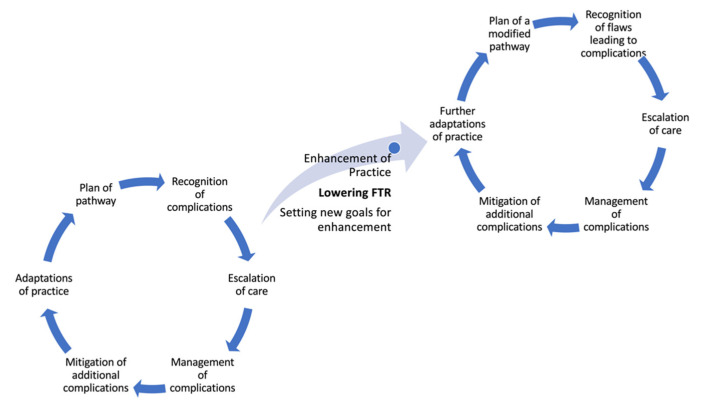
The proposed six-step approach that takes into consideration the steps of the PDSA (Plan-Do-Study-Act) cycle of quality improvement science to address and lower the failure to rescue (FTR) rate. Starting each cycle from the plan of pathway and continuing to the next five steps leads to the enhancement of practice and lowering of FTR. Nonetheless, as a cycle closes, new goals for improvement are set and a new cycle of improvement opens, thus driving a never-ending quality improvement process.

Two main systematic approaches have been proposed to enable and secure successful patient rescue following a major complication. The first one was proposed by Hatchimonji et al. [29] and included a two-step strategy: (a) recognition of complications and (b) management of complications. Despite its relatively simple and one-dimensional approach, it represents the first attempt to establish a systematic methodology for the management of postoperative morbidity and reduce FTR. A more advanced and feasible strategy has recently been proposed by Gross et al. [30]. This strategy includes four steps: (a) early recognition of a rescuable complication, (b) timely escalation of care, (c) effective management, and (d) mitigation of additional complications. In fact, this is a much more efficient and feasible approach that enables the reduction of FTR along with the enhancement of quality outcomes. In this review, we propose a differentiated six-step approach that takes into consideration the steps of the PDSA (Plan-Do-Study-Act) cycle of quality improvement science (Figure 4).

Our roadmap to a successful rescue is based on Gross’s approach and includes the following steps:

(1) Plan of a protocolized pathway;
(2) Early recognition of a complication;
(3) Adequate escalation of care;
(4) Effective management of the complication;
(5) Mitigation of additional complications;
(6) Review of potential flaws in the pathway and appropriate adaptations of practice.

### 3.7. Plan

This step involves the identification of goals, defining success metrics and putting a protocolized plan into action. Such quality goals or metrics include the time until extubation, goal-directed fluid administration, the limitation of opioid administration, and the implementation of additional enhanced recovery strategies.

### 3.8. Early Recognition of Complications

The timely recognition of postoperative complications requires active and vigilant monitoring because slow clinical deterioration usually precedes complications, thus offering a chance for early intervention [31]. Crucial pillars in this step are the experience and education of staff, including both physicians and nurses.

### 3.9. Escalation of Care

Following the early recognition of complications, the phase of escalation of care begins. The main goals are to (a) communicate a patient’s deterioration with the senior clinician, (b) transfer the patient from the ward to ICU, if needed, for enhanced monitoring, (c) initiate a thorough diagnostic work-up, and (d) consult with specialists relevant to the disease of interest. The timely escalation of care is crucial for reducing mortality and successfully rescuing surgical patients presenting complications [32].

### 3.10. Effective Management

The management of a patient with a complication occurs in two distinct phases: (a) hemodynamic and respiratory stabilization and (b) definitive treatment of the underlying pathology. Hemodynamic stabilization and adequate tissue perfusion represent the primary endpoints of this phase of care and should be guided by attending physicians. The second phase includes the identification and definitive treatment of the underlying pathology. Patients with severe postoperative cardiogenic morbidity/shock require invasive monitoring, appropriate inotropic treatment, and, potentially, an intra-aortic balloon pump, a temporary ventricular assist device (VAD), or extracorporeal membrane oxygenation (ECMO) [33].

### 3.11. Mitigation of Additional Complications

The presence of multiple complications following cardiac surgery is an independent risk factor associated with an increased risk of mortality [34]. In fact, the greater the number of postoperative complications is, the higher the risk for postoperative mortality is [31]. Ghaferi and colleagues [28] conducted a retrospective study that incorporated data from 266,101 patients who underwent high-risk general, vascular, or thoracic surgery. According to their findings, there is a linear correlation between number of complications and FTR rates [28]. In another study, which included 9532 cardiac surgery patients, a correlation was demonstrated between number of complications and FTR rates [31]. Consequently, it is crucial to mitigate additional complications early in close collaboration with the associated specialties.

### 3.12. Review of Potential Flaws

Even in the most effective and advanced settings, there are opportunities for the enhancement of quality and outcomes. There are two main sources driving this change: (1) the systematic monitoring of outcomes and (2) emerging evidence on new methodologies, techniques, or equipment. The systematic monitoring of outcomes is crucial in identifying potential room for improvement or flaws in the protocolized pathway. There are certain measures that should be undertaken to establish this environment of change. These include the following:

(1) Hiring data managers. These are professionals devoted to data mining and analysis in collaboration with statisticians. This is the first step that allows for further review of the effectiveness of current practices.
(2) Performing audits. Audits can take place in-person or virtually. A random selection of cases from the most recent 6 to 12 months is examined for the correctness and completeness of data.
(3) Performing a phase of care mortality analysis (POCMA). This is a case review of patients who died during the perioperative pathway. These cases are discussed either among faculty members or in the context of a society meeting. The goal is to identify the phase of care at which the primary event leading to death occurred. This is a method adopted by the Michigan Society of Thoracic and Cardiovascular Surgeons—Quality Collaborative (MSTCVS-QC) to evaluate all surgical mortality events in Michigan that relies on the concept that “all cardiac surgical deaths are initiated by a seminal event that triggers a cascade of deterioration culminating in death” [35]. In fact, MSTCVS-QC, historically led by Dr. Prager, has paved the way for numerous advances and novelties in the direction of raising standards in patients’ safety [36].
(4) Planning quarterly meetings. These should serve as a forum for scientific discussion among surgeons on their outcomes. Data should be presented in an unblinded manner, allowing for the identification and discussion of variations in inter-institutional outcomes. Overall, these meetings exemplify how a quality improvement program can be structured with a collaborative nature. Data should also be compared with STS outcomes.

### 3.13. FTR in Intensive Cardiovascular Care

Historically, FTR was developed to assess the quality of clinical outcomes in surgical populations and was extensively implemented in cardiac surgery. However, FTR seems to also have an important role in cardiovascular intensive care and in cardiology, thus providing additional value. The concept behind this observation is that these disciplines manage complex cases and use several interventional procedures and their outcomes are highly heterogeneous among different institutions [33]. In fact, according to a statewide analysis of FTR following percutaneous coronary intervention (PCI), the FTR rate ranged from 11.6% to 15.0% with significant differences between high- and low-volume centers [33]. Perhaps, more studies are necessary to fully validate and establish FTR as a quality metric in interventional cardiology and cardiovascular intensive care.

### 3.14. FTR in Other Surgical Specialties

Following its success in providing enhanced insight into the quality of a practice, FTR was rapidly implemented in a wide spectrum of surgical specialties. First of all, except from adult cardiac surgery, FTR has also been implemented in pediatric cardiac surgery (FTR: 4.1%) [10] and heart transplantations [37]. In addition, FTR has also been validated for patients undergoing thoracic (FTR: 16.3%) [38], major abdominal (FTR: 23.5%) [39,40,41], and vascular surgeries (FTR: 9.6–21.5%) [42]. As demonstrated, FTR rates in these different disciplines are similar to those observed in cardiac surgery. Nonetheless, the bias of an inadequate FTR definition also exists in these specialties.

## 4. Discussion

The present study attempted to systematically summarize the current evidence provided by the literature on the emerging role of FTR as a quality metric in cardiac surgery and cardiovascular intensive care. In this context, the current review represents a valuable source of information regarding the evolution and expanding implementation of FTR in clinical practice for all physicians and surgeons willing to establish quality improvement practices. In fact, in the previous sections, we discussed not only the evolution of the FTR metric but also the different factors affecting it, and we demonstrated a stepwise roadmap for reducing the FTR rate.

The importance of FTR as a quality metric can be easily highlighted by comparing the generally low and homogeneous mortality rates in Table 1 with the respective higher FTR rates. Since cardiac surgery is generally associated with a low mortality rate, we need a different quality metric to guide our efforts for improvement, and FTR seems to be the most suitable candidate for this role. Nonetheless, as demonstrated in the previous sections, there is currently significant heterogeneity in the literature regarding the number of complications included in the definition of FTR. Over the years, there has been a continuous trend to lower this number and include only major complications. In this context, there has been a decrease from 17 and 10 complications in 2013 [20] and 2014 [13], respectively, to 4 major complications in 2022 [17]. These are stroke, renal failure, reoperation, and prolonged ventilation [17]. Perhaps, this heterogeneity in the definition of FTR contributed to a significant range of FTR percentages, between 5.4% and 19.8%. This evidence demonstrates the urgent need for a consensus on the definition and coding of FTR to generate homogenous, comparable, and robust real-life data that will drive future quality improvement initiatives.

Cardiac surgery has always spearheaded efforts for surgical advances, and FTR was not an exception. In fact, surgeons in different specialties have started to implement FTR in their practices as a quality measure [38,39,43]. However, cardiac surgery has been, once more, at the forefront of experimentation, and recently, the use of FTR has diverged from assessing only mortality to include also severe morbidity [33]. The main reason behind this diversion is that there are certain postoperative complications that might not necessarily lead to death but contribute to an unacceptable postoperative quality of life for patients. Such examples are patients with newly acquired renal deficiency, needing dialysis, or patients postoperatively presenting with stroke. In these cases, where the failure to rescue refers to morbidity instead of mortality, a new term is used, FTR-Mb, where “Mb” represents morbidity. Nonetheless, the reasoning behind this concept is primarily based on preoperative risk stratification. For instance, patients with severe bilateral carotid stenoses undergoing emergent cardiac surgery have a high risk for stroke, and patients with chronic renal failure have an increased risk for acute renal injury [30]. In such cases, the success of rescue might not be possible regarding morbidity, and measuring FTR-Mb might be out of context. Nonetheless, FTR-Mb can be a very useful tool when attempting to assess and compare clinical outcomes and quality in terms of postoperative morbidity among different institutions.

FTR is rapidly becoming an important quality metric for cardiac surgery and cardiovascular intensive care. Special remarks should be made regarding two important pillars affecting FTR. The first pillar includes all factors that affect and either increase or lower the FTR. These are divided into either hospital- or patient-related factors. Accurate preoperative risk stratification is crucial in order to assess and manage patient-related factors and to plan the perioperative treatment pathway. Hospital-related factors are more complex and include the level of expertise and education of the staff, the participation of residents or attending physicians in the perioperative management, the volume of patients that the institution treats, and the presence of adjacent specialties capable of rapidly managing newly emergent and potentially mortal complications. The second pillar is associated with the adoption of a stepwise approach to reduce FTR. In the present review, we proposed such a roadmap aiming to help clinicians in their daily duties. In this context, the FTR metric should target new, severe complications for which there are known treatment interventions. Optimizing patient rescue requires the early planning of a protocolized treatment pathway, the early recognition of complications and escalation of care, the mitigation of additional complications, and, finally, the recognition of potential flaws with respective adaptations in clinical practice. Following such a strategy, FTR implementation allows for the enhanced allocation of resources and for building a safe culture, thus leading to better outcomes.

The limitations of the present review are mainly associated with the limitations of the included studies. Most of the studies were retrospective and no RCT was identified through the literature search, thus posing a certain limitation to this study. Furthermore, the incorporated studies may have biases related to participant selection and performance. In addition, the differences among institutions regarding the definition of FTR, treatment protocols, selection criteria, and perioperative management pose several limitations. On the other hand, the strengths of the present review include (1) the clear literature search and data-extraction protocol, (2) the well-specified inclusion/exclusion criteria, (3) the literature search in three databases, (4) the quality assessment of the included studies, and the detailed presentation of the outcomes.

## 5. Conclusions

FTR is evolving into a crucial quality metric for cardiac surgery and cardiovascular intensive care. However, to be effectively used, the FTR metric should focus on new, life-threatening complications for which there are known interventions. Consequently, more efforts are needed to provide a more robust definition of FTR. There are several factors that affect FTR, and the adoption of a stepwise roadmap in clinical practice is necessary to reduce the FTR rate. Further studies are necessary to further validate FTR, assess its intra-institutional heterogeneity, and also assess the potential value of FTR for morbidity (FTR-Mb).

## Figures and Tables

**Table 1:** Baseline characteristics of the studies and patients that were included in the present review. Abbreviations: FTR = failure to rescue; NOS = Newcastle–Ottawa Scale; R = retrospective; STS = Society of Thoracic Surgeons; MSTCVS-QC = Michigan Society of Thoracic and Cardiovascular Surgeons—Quality Collaborative; NRD = Nationwide Readmissions Database; N/A = not available; *n* = number.

Study ID, Year	Country	Study Design	Study Population, *n*	Complications, *n* (%)	Mortality, *n* (%)	FTR, %	NOS
Ahmed 2014 [13]	Canada	R	4978	834 (16.8)	180 (3.6)	19.8	7
Dewan 2021 [14]	USA	R	75,851	43,437 (63.4)	6151 (9)	13.4	7
Dewan 2022 [15]	USA	R	103,757	31.1–36.7%	1394 (2)	5.4–15.5	7
Edwards 2016 [16]	USA	R–STS	604,700	78,611 (13)	8228 (1.4)	10.5	7
Kurlansky 2022 [17]	USA	R–STS	1,058,138	Ν/A	27,045 (2.6)	14.7	7
Likosky 2022 [18]	USA	R	83,747	30,265 (36)	1648 (2)	11.6	7
Milojevic 2021 [19]	USA	R–MSTCVS-QC	62,450	16.3–21.3%	1418 (2.3)	8.3–12.7	7
Reddy 2013 [20]	USA	R–STS	45,904	19.4–22.9%	2.6%	6.6–13.5	7
Sanaiha 2019 [21]	USA	R–National Inpatient Sample	2,012,104	36%	2%	N/A	7
Shahian 2018 [1]	USA	R–STS	N/A	N/A	N/A	N/A	6
Verma 2023 [22]	USA	R–NRD	454,506	32,537 (7.2)	7669 (1.7)	16.7	7

## Data Availability

The data that support the findings of this study are available from the corresponding author, upon reasonable request.

## Supplementary Materials

The following supporting information can be downloaded at: https://www.mdpi.com/article/10.3390/jcm12144876/s1, Table S1: PRISMA CHECKLIST.
